# miR-195-5p Suppresses KRT80 Expression Inducing Cell Cycle Arrest in Colon Cancer

**DOI:** 10.3390/cancers17132183

**Published:** 2025-06-28

**Authors:** Emanuele Piccinno, Viviana Scalavino, Nicoletta Labarile, Giusy Bianco, Raffaele Armentano, Gianluigi Giannelli, Grazia Serino

**Affiliations:** National Institute of Gastroenterology S. De Bellis, IRCCS Research Hospital, Via Turi 27, 70013 Castellana Grotte, Italy; emanuele.piccinno@irccsdebellis.it (E.P.); viviana.scalavino@irccsdebellis.it (V.S.); nicoletta.labarile@irccsdebellis.it (N.L.); giusy.bianco@irccsdebellis.it (G.B.); raffaele.armentano@irccsdebellis.it (R.A.); gianluigi.giannelli@irccsdebellis.it (G.G.)

**Keywords:** CRC, miR-195-5p, KRT80, keratins, cell cycle

## Abstract

Background/Objectives: Keratins form a crucial component of the epithelial cytoskeleton, playing an essential role in maintaining tissue architecture and coordinating key cellular functions. KRT80 is a type II keratin that has emerged as an oncogenic driver in several malignancies, yet its involvement in colorectal cancer (CRC) remains unclear. Here, we investigated the molecular interaction between miR-195-5p, KRT80 expression, and CRC growth. Methods: Potential miR-195-5p binding sites in the KRT80 3′-UTR were identified through the use of integrated bioinformatic analyses, while publicly available datasets confirmed a significant overexpression of KRT80 in CRC tissues compared to normal mucosa. This finding was further validated through the use of mRNA and protein analysis in paired tumor and adjacent normal samples from CRC patients. Results: Functional assays involving CRC cell lines showed that transfection with miR-195-5p mimics led to a significant downregulation of KRT80 expression, reflecting the effects of direct KRT80 silencing by siRNA. Both molecular approaches induced G_1_-phase cell cycle arrest, concomitantly with reductions in G_2_/M populations. Furthermore, the in vivo delivery of miR-195-5p mimics in a mouse model of colitis-associated CRC resulted in a significant reduction in *Krt80* expression in the colon. Conclusions: Collectively, our results reveal that miR-195-5p negatively regulates KRT80 expression, contributing to its tumor-suppressive activity in colorectal cancer and highlighting a molecular mechanism with potential therapeutic relevance.

## Simple Summary

Cytokeratin 80 (KRT80) is a protein type belonging to the keratin family, which has been correlated with more aggressive tumor phenotypes, higher rates of proliferation, enhanced migratory and invasive capabilities, and poorer clinical outcomes in different cancers. Although KRT80 may act as a mediator of oncogenic signaling, the molecular mechanisms in colorectal cancer (CRC) development still need to be evaluated. Here, we aimed to investigate the miR-195-5p molecular mechanism related to the modulation of KRT80 that underlies its role in CRC, emphasizing the potential properties of this miRNA for therapy. In summary, this study provides evidence that KRT80 is significantly overexpressed in CRC patients. Moreover, miR-195-5p effectively restores the KRT80 expression level in CRC cell lines and azoxymethane (AOM)/dextran sulfate sodium (DSS)-treated mice, highlighting its regulatory role. Additionally, the critical oncogenic properties of KRT80 were demonstrated that, once silenced, suppressed tumor growth. These findings further reinforce the clinical potential of miR-195-5p in regard to affecting keratin expression and influencing CRC progression.

## 1. Introduction

Cellular interactions ensure the stability, integrity, and function of tissues and are involved in several physiological and pathological processes. Cytokeratin 80 (KRT80) is a protein type belonging to the keratin family, which consists of fibrous structural proteins that form the intermediate filaments in epithelial cells that are crucial for the structure and activity of the cytoskeleton, providing mechanical support, protecting cells from stress, and mediating signal transduction [1,2]. Within this framework, the aberrant expression of a keratin member has been linked to various malignant disorders, including CRC [2,3,4]. The human type II keratin, KRT80, is an integral intermediate filament-forming component that is involved in many biological functions; it is located on the epithelial cell surface, close to the desmosome complex during the early stage of differentiation, and is embedded throughout the cytoplasm in epithelial cells [5]. In the last few years, KRT80 activity has been investigated due to its peculiar features related to cancer mechanisms and patient outcomes. KRT80 could distinguish between good and poor survival outcomes in gastric cancer (GC) patients and is an independent risk factor for the prognosis of patients with ovarian cancer [6,7,8]. A higher expression of KRT80 has also been correlated with the promotion of the epithelial–mesenchymal transition in hepatocellular carcinoma (HCC) cell lines, affecting the PI3K/AKT signaling pathway [9]. In lung adenocarcinoma, KRT80 knockout has been reported to inhibit proliferation, suppressing the transition of cancer cells from the G1 to the S phase [10]. Moreover, in endocrine-resistant breast cancer, the epigenetic activation of KRT80 by Sterol Regulatory Element-binding Protein 1 (SREBP1) drives a type of cytoskeletal reprogramming that promotes migration, invasion, and chemotherapeutic resistance [11]. Additional studies further support the oncogenic properties of KRT80 in terms of tumor biology; for instance, in esophageal squamous cell carcinoma, KRT80 overexpression enhances proliferation, invasiveness, and lymph node metastasis [12]. It has also been reported that the overexpression of KRT80 induced changes in CRC cell morphology, which shifted towards a mesenchymal phenotype, triggering the AKT cascade and facilitating cell survival and motility [13].

In addition, miRNAs are small noncoding single-stranded RNA molecules involved in several molecular processes, thanks to their ability to regulate gene expression, acting on mRNA sequences at the post-transcriptional level [14]. Owing to their properties, miRNA deregulation may lead to the development of cancerous conditions [15,16,17] and many studies have highlighted their effectiveness in the prevention and suppression of neoplasms, affecting keratin expression. It has been shown that miR-365-3p/KRT16 signaling is involved in the tumorigenesis and drug resistance of oral squamous cell carcinoma, modulating the β5-integrin/c-Met pathway [18]. Shi and co-workers found that miR-642a-5p exerted a negative effect on KRT19 expression, inhibiting pancreatic cancer progression via the Wnt/β-catenin pathway [19]. Other recent reports have revealed a miR-485-5p/KRT17-driven signaling cascade involving integrin activation and downstream FAK, Src, ERK, and β-catenin pathway modulation, which sustained cancer stemness properties and conferred resistance to cisplatin in oral squamous cell carcinoma [20]. In regard to gastric carcinoma, miR-4268 suppressed tumor progression by directly targeting KRT80 to inhibit PI3K/AKT/JNK signaling [6].

In our previous work, we highlighted that miR-195-5p had the ability to repress the expression of pinin (PNN), a desmosome member, aberrantly expressed in CRC, which is involved in multiple cellular processes that interact and cooperate with KRT8 and KRT19, whose levels were also indirectly modulated [21]. Moreover, we have also shown that, in vitro and in vivo, the gain in miR-195-5p clearly suppressed CRC progression, reducing the expression of KRT23, a keratin with pro-oncogenic properties [22].

Here, our aim is to investigate and characterize the miR-195-5p molecular mechanism related to the modulation of KRT80 that underlies the key role of keratins in the CRC outcome, emphasizing the potential properties of this miRNA for CRC therapy.

## 2. Materials and Methods

### 2.1. Human Paired CRC and Non-Tumor Tissues

Sixty tissue samples, including both tumor and adjacent normal sections, were obtained from 60 patients with colorectal cancer (CRC), at the National Institute of Gastroenterology “S. de Bellis” in Castellana Grotte, Bari, Italy. The inclusion criteria were: (1) patients older than 18 years; (2) diagnosed with colon adenocarcinoma; and (3) no neoadjuvant chemotherapy. All the participants provided written informed consent. The study adhered to the Declaration of Helsinki and was approved by the local institutional ethics review board (Istituto Tumori Giovanni Paolo II, Bari, Italy, n° 379/2020 on 16 September 2020). The tissue sections were stained with hematoxylin and eosin and subsequently evaluated by a pathologist to confirm their suitability and to assess their morphological and pathological characteristics.

### 2.2. Immunohistochemistry of KRT80 in FFPE Human Samples

Before immunostaining, 60 formalin-fixed paraffin-embedded (FFPE) tissues were sectioned, with a thickness of 3 µm, and mounted on Apex Bond IHC slides (Leica Biosystems, Buffalo Grove, IL, USA). The sections were deparaffinized and processed using a BOND III automated immunostainer (Leica Biosystems, Buffalo Grove, IL, USA), with the anti-KRT80 primary antibody (ab222325, Abcam, Cambridge, UK; 1:100 dilution). After staining, the sections were counterstained with hematoxylin. Antigen retrieval was performed using the BOND Epitope Retrieval Solution 2 (Leica Biosystems), with a citrate buffer (pH 6), and the Bond Polymer Refine Detection Kit (Leica Biosystems) served as the chromogen reagent. A pathologist then scored the immunohistochemical staining based on both the extent and intensity of the biomarker, as follows: 0 (no staining/negative), 1 (weak expression), 2 (moderate expression), and 3 (strong expression).

### 2.3. Cell Cultures

For all the investigations, two human colon cell models were employed. HCT116 and HT29 cells were obtained from ATCC (American Type Culture Collection, Manassas, VA, USA) and were maintained in Dulbecco’s Modified Eagle Medium (DMEM, Thermo Fisher Scientific, Waltham, MA, USA), supplemented with 10% heat-inactivated fetal bovine serum (FBS, Thermo Fisher Scientific, Waltham, MA, USA), 10 mM of HEPES (Sigma-Aldrich, St. Louis, MO, USA), 1 mM of sodium pyruvate (Sigma-Aldrich, St. Louis, MO, USA), and 1% streptomycin/penicillin (Thermo Fisher Scientific, Waltham, MA, USA). All the cell lines were incubated at 37 °C in an atmosphere containing 5% CO_2_.

For the RNA extraction, the cells were seeded into 12-well plates, at a density of 1.5 × 10^5^ cells per well, while for the protein isolation, the cells were plated in 6-well plates, at densities of 2.5 × 10^5^. Upon reaching confluence, the cells were transiently transfected with synthetic miR-195-5p mimic molecules with or without FAM (Life Technologies, Carlsbad, CA, USA) at concentrations of 30 nM and 50 nM or siKRT80 (s44669 and s44670, Life Technologies, Carlsbad, CA, USA), using the TransIT-TKO Transfection Reagent (Mirus Bio LLC, Madison, WI, USA), according to the manufacturer’s protocol. To assess the cell cycle activity, the cells were initially seeded in 6-well plates and then transfected. Each experiment featured a mock control in which the cells were transfected solely with the TKO reagent without nucleic acid molecules.

### 2.4. RNA Extraction and RT-PCR of Cell Cultures, Human and Mouse Sections

The total RNA from the cell cultures (twenty-four hours post-transfection) of human and mice tissues was extracted using the TRIzol reagent (Invitrogen by Thermo Fisher Scientific, Waltham, MA, USA), resuspended in ribonuclease-free water, and quantified using the NanoDrop ND-2000 Spectrophotometer (Thermo Fisher Scientific, Waltham, MA, USA).

To analyze the expression of miR-195-5p after the transfections, the RNA was reverse transcribed using a TaqMan Advanced miRNA cDNA Synthesis Kit (Thermo Fisher Scientific, MA, USA). Then, RT-PCR was performed, using a final volume of 20 μL and a CFX96 System (Bio-Rad Laboratories, CA, USA), with TaqMan Advanced miRNA assays and the TaqMan Fast Advanced Master Mix (Thermo Fisher Scientific, MA, USA). The data was normalized using miR-26a-5p as a reference. The relative expression was calculated using the 2^−ΔΔCt^ formula. The RNA was then reverse transcribed using the iScript Reverse Transcription Supermix (Bio-Rad Laboratories, CA, USA), following the manufacturer’s guidelines. To assess the KRT80 expression, a quantitative real-time PCR was performed using the CFX96 System (Bio-Rad Laboratories, Hercules, CA, USA), with the SsoAdvanced Universal SYBR Green Supermix (Bio-Rad Laboratories, Hercules, CA, USA) and the QuantiTect Primer Assay for KRT80 and GAPDH (Qiagen, Hilden, Germany). The Murine QuantiTect Primer Assay for *Krt80* (Bio-Rad Laboratories, Hercules, CA, USA) and *Gapdh* (Qiagen, Hilden, Germany) were also used. The expression levels of the target gene and the housekeeping gene were determined from four independent experiments, with GAPDH serving as the normalization standard; relative gene expression was computed using the 2^−ΔCt^ or 2^−ΔΔCt^ method.

### 2.5. Protein Extraction and Western Blot Analysis

Seventy-two hours after transfection, the total protein was extracted using the T-PER Tissue Protein Extraction Reagent (Thermo Fisher Scientific, Waltham, MA, USA), supplemented with a cocktail of proteinase inhibitors (Sigma-Aldrich, St. Louis, MO, USA). The protein concentration was measured using the Bradford colorimetric assay (Bio-Rad Laboratories, Richmond, CA, USA). An equal amount of protein from each sample was heat denatured at 100 °C for 5 min in reducing Laemmli Sample Buffer (Bio-Rad Laboratories, Hercules, CA, USA), and was loaded onto 4–20% Mini-PROTEAN TGX Stain-Free Protein Gels (Bio-Rad Laboratories, Hercules, CA, USA) and subsequently transferred onto PVDF membranes, with a 0.2 μm pore size (Bio-Rad Laboratories, Hercules, CA, USA). The membranes were processed using an automated iBind Flex Western Device (Thermo Fisher Scientific, Waltham, MA, USA), with the appropriate primary and secondary antibodies. The signal intensities were detected using the Chemidoc System (Bio-Rad Laboratories, Hercules, CA, USA), and the images were analyzed using Image Lab Software version 5.2.1 (Bio-Rad Laboratories, Hercules, CA, USA) and quantified using ImageJ Software 1.54d. Moreover, β-tubulin was used as the internal control for normalization.

In these experiments for protein detection, the primary antibodies included: rabbit polyclonal anti-KRT80 (Abcam ab222325, Cambridge, UK; 1:1000 dilution) and mouse monoclonal β-tubulin (sc-166729, Santa Cruz Biotechnology, Inc., Heidelberg, Germany; 1:1000 dilution). The secondary antibodies used were Goat Anti-mouse IgG-(H+L)–HRP conjugate (170-6516, Bio-Rad Laboratories, CA, USA; 1:500 dilution) and Goat Anti-rabbit IgG-(H+L)–HRP conjugate (#31466, Invitrogen, Carlsbad, CA, USA; 1:2500 dilution).

### 2.6. Cell Cycle

HCT116 and HT29 cells were seeded in 6-well plates, at 2 × 10^5^ cells per well, and then transfected. After 24 h of transfection, 100 ng/mL of nocodazole was added for 24 h to induce mitotic arrest. Three hours after nocodazole block release in complete media, the cells from each well were detached and centrifuged. The pellets were then washed with PBS and fixed by adding cold 70% ethanol, for 3 h, at −20 °C. The fixed cells were then centrifuged, the supernatant discarded, washed in PBS, and 200 μL of the Muse^®^ Cell Cycle Kit (Cytek Biosciences, Fremont, CA, USA) was added. After 30 min of incubation at room temperature in the dark, the samples were analyzed using the Muse^®^ Cell Analyzer, according to the manufacturer’s instructions.

### 2.7. Immunofluorescence in Mouse Samples

Three μm thick sections of FFPE mouse colon were deparaffinized, and antigen retrieval was achieved by incubating the slides in citrate buffer (pH 6) in a water bath at 98 °C for 30 min. The slides were then treated with a 0.5% Triton X-100 solution in 1× PBS for 10 min at room temperature, followed by a blocking step, in 5% BSA in PBS for 1 h at room temperature. Next, the sections were incubated overnight at 4 °C with a primary antibody against cytokeratin 80 (16835-1-AP, Proteintech., Manchester, United Kingdom; dilution 1:100), and, subsequently, with a secondary antibody, chicken secondary antibody chicken anti-Rabbit IgG (H + L) Alexa Fluor 594 (A-21442, Invitrogen, Carlsbad, CA, USA, dilution 1:200) for 1 h. Finally, the slides were mounted with ProLong Gold Antifade Mountant with DAPI (Thermo Fisher Scientific) and a glass coverslip, and images were captured using a fluorescence microscope (Eclipse Ti2, Nikon Inc., Melville, NY, USA), equipped with DAPI and FITC filters and a 20× objective.

### 2.8. Bioinformatic and Statistical Analyses

The differential expression of the KRT80 gene and protein in CRC versus matched normal tissues was examined using the University of ALabama at Birmingham CANcer data analysis Portal (UALCAN) (https://ualcan.path.uab.edu/, accessed on 27 March 2024) [23]. To identify potential gene targets of miR-195-5p, the miRabel algorithm (http://bioinfo.univ-rouen.fr/mirabel/, accessed on 28 February 2024) [24], miRWalk 3.0 (http://mirwalk.umm.uni-heidelberg.de/, accessed on 28 February 2024) [25], Tarbase (https://dianalab.e-ce.uth.gr/html/diana/web/index.php?r=tarbasev8/index, accessed on 15 March 2024) [26], and miRmap (https://mirmap.ezlab.org/, accessed on 15 March 2024) [27] were employed. The KRT80 biological interactions in regard to tumoral mechanisms were investigated by performing an analysis using Genemania, (https://genemania.org/, accessed on 24 April 2024) [28], a prediction tool that constructs a composite gene–gene functional interaction network.

The data from the analyses were processed using GraphPad Prism version 10.0.2, expressed as the mean ± SEM. Statistical significance was determined via a two-tailed Student’s *t*-test on results from at least three independent experiments, with *p* < 0.05 being considered significant.

## 3. Results

### 3.1. miR-195-5p as KRT80 Potential Regulator

With the aim of identifying new potential genes that encode for keratin components targeted by miR-195-5p, we carried out a bioinformatic analysis to predict the miRNA–mRNA interactions.

Our results, based on overlapping data from different databases, indicated two potential binding sites in the 3′UTR of the KRT80 gene, suggesting that it could be a candidate target for miR-195-5p (Table S1).

### 3.2. KRT80 Levels in Patients with Colon Cancer

We have previously demonstrated the lowered expression of miR-195-5p in CRC [29]. To investigate whether KRT80 expression could be aberrant in patients affected by CRC we evaluated its levels using UALCAN, an online tool that analyzes the cancer OMICS data from The Cancer Genome Atlas (TCGA) and the Clinical Proteomic Tumor Analysis Consortium (CPTAC).

**Figure 1:**
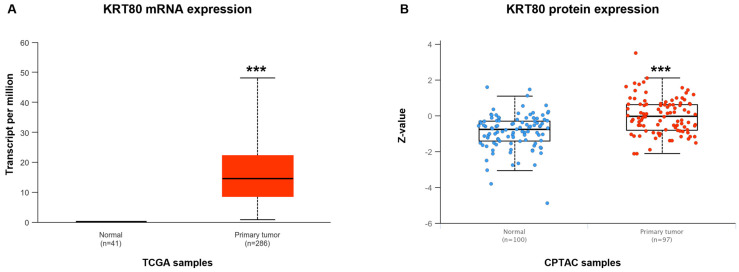
KRT80 mRNA (**A**) and protein (**B**) expression in normal colon and CRC tissues. UALCAN software version 1.1.1 revealed a marked and significant KRT80 upregulation in cancerous conditions; *** *p* < 0.0001.

The data derived from the analysis of CRC patients underlined KRT80 overexpression in tumoral tissue compared to healthy controls at both mRNA and protein levels (*p* < 0.0001; Figure 1).

**Figure 2:**
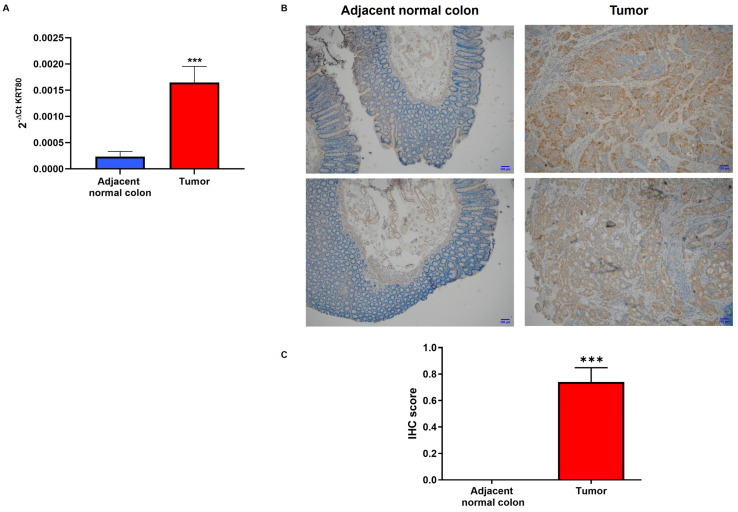
KRT80 levels in CRC patients. Surgical resections from 60 patients that included cancerous tissue and peritumoral colon samples were used for the molecular assay. (**A**) KRT80 mRNA quantification using PCR analysis showed a strong increase in CRC patients compared to non-cancerous controls; *** *p* < 0.0001. (**B**) IHC acquisition at 4× magnification showing a clear, more intense positivity in tumoral sections. (**C**) Immunoreactivity score derived from the quantification of IHC preparations in terms of KRT80 expression in healthy and diseased tissues; *** *p* < 0.0001.

The bioinformatic analysis was also biologically validated by evaluating the KRT80 mRNA and protein expression in the tissue of patients who underwent surgery in our research hospital. The examined samples (*n* = 120) included tumoral and adjacent normal mucosa from 60 CRC patients. The qPCR assay revealed a significant difference between the KRT80 levels in the tissue of 30 CRC patients and the control mucosa (*p* < 0.0001; Figure 2A). These data were also validated using immunohistochemistry (IHC) staining performed on a different cohort of 30 CRC patients, highlighting once more that KRT80 expression was found only in the tumor portion (*p* < 0.0001; Figure 2B). Moreover, based on the strength of the positive intensity obtained in regard to the IHC preparations, we established an immunoreactivity score, assigning a value between 0 and 3. The amount of positive staining further confirmed the increased levels of KRT80 in CRC patients (Figure 2C).

### 3.3. miR-195-5p Controls’ KRT80 Expression

**Figure 3:**
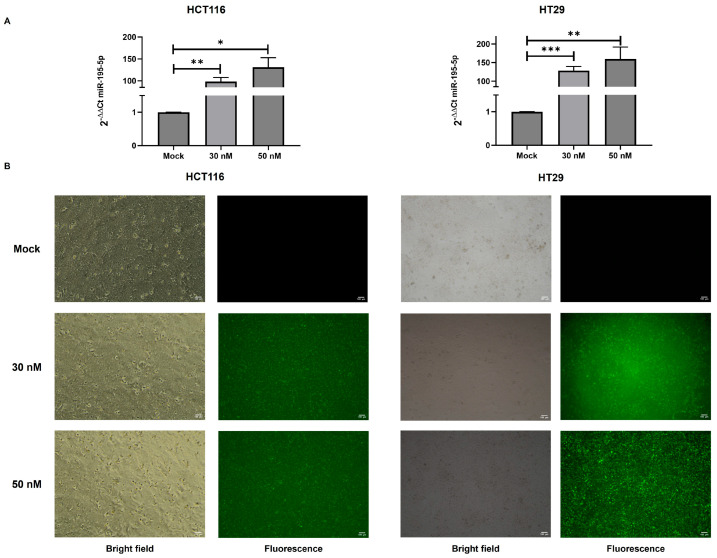
Transfection efficiencies of HCT116 and HT29 cell lines. (**A**) The expression of miR-195-5p after transfection was significantly increased in both cell lines at 30 nM and 50 nM concentrations compared to the mock control. Expression data are representative of four independent experiments (*n* = 4; mean ± SEM); * *p* < 0.05, ** *p* < 0.001, *** *p* < 0.0001. (**B**) HCT116 and HT29 cells were transfected with FAM-labeled miR-195-5p mimic at 30 nM and 50 nM concentrations. Bright field (**left**) and fluorescence (**right**) representative images for the mock and transfected conditions were acquired at 10× magnification.

In order to characterize the in vitro effectiveness of miR-195-5p on KRT80 expression, we performed transient transfection with miR-195-5p mimic molecules in the human colonic cancer cells, HCT116 and HT29. We first assessed the transfection efficiency in all the cell lines, analyzing the expression of miR-195-5p after transfection. A significant increase in miR-195-5p levels was observed after transfection at a concentration of 30 nM and 50 nM in both cell lines (*p* < 0.05; Figure 3A). To further validate these findings, we examined the intracellular uptake and distribution of miR-195-5p mimic molecules using FAM-labeled miR-195-5p mimic molecules (Figure 3B).

**Figure 4:**
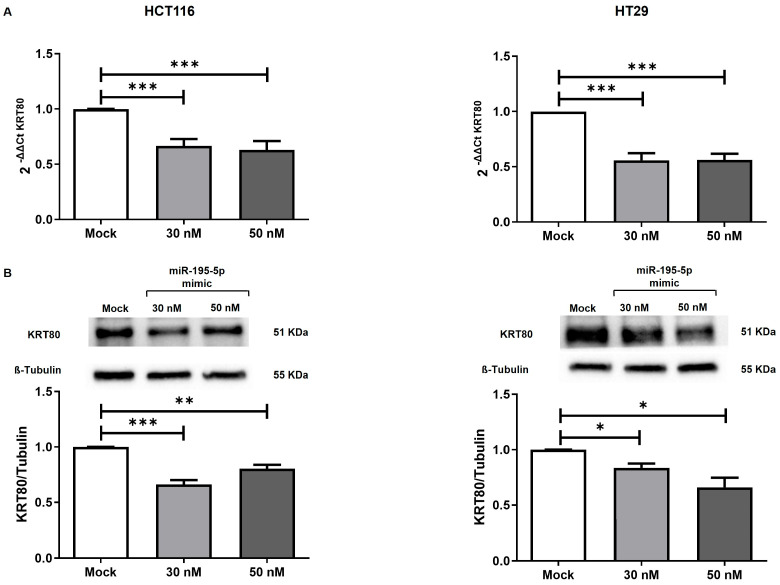
KRT80 expression in CRC transfected cell lines. HCT116 (**left**) and HT29 (**right**) were treated with synthetic compounds of miR-195-5p mimic highlighting a KRT80 mRNA (**A**) downregulation compared to the mock control. KRT80 values were normalized in regard to GAPDH expression and the histograms are descriptive of at least four experiments (mean ± SEM); *** *p* < 0.0001. (**B**) KRT80 expression at protein level in an miR-195-5p transfected cell. The effect of miR-195-5p mimic molecules on the decreased KRT80 expression was observable in both cell lines, as well as at both mimic concentrations. KRT80 levels were normalized in regard to the values of the β-tubulin housekeeping protein and then assessed by dividing the normalized transfected sample values based on the normalized mock control sample values; *n* = 4; * *p* < 0.01; ** *p* < 0.001; *** *p* < 0.0001.

The induction of miR-195-5p at 30 nM and 50 nM concentrations led to a remarkable decrease in KRT80 mRNA levels (*p* < 0.0001; Figure 4A) in HCT116 and HT29 cell lines. A reduction in KRT80 protein expression was also observed after transfection in both cell lines (*p* < 0.05; *p* < 0.001; *p* < 0.0001; Figure 4B).

### 3.4. In Vivo Evaluation of miR-195-5p Effect on KRT80 Expression

To fully elucidate the positive effects of miR-195-5p on the modulation of KRT80, we investigated the KRT80 expression in colon segments we had previously obtained from AOM/DSS CRC mice [29]. Briefly, Male C57BL/6 mice (7 weeks old; 19–25 g) were randomly assigned to either a vehicle control group or an miR-195-5p treatment group. Both groups underwent the cyclic administration of AOM and DSS, followed by a 15-day recovery period with distilled water. Mice in the treatment group received miR-195-5p injections twice weekly, until the time of sacrifice, when the medial and distal colon portions of the mice were collected.

**Figure 5:**
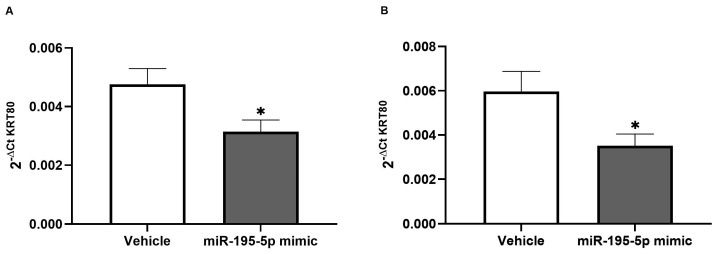
The miR-195-5p mimic as modulator of Krt80 expression in the CRC mice model *(n* = 14 mice/group). In vivo treatment with miR-195-5p mimic molecules resulted in a significant reduction in KRT80 expression in the medial portion (**A**), as well as the distal colon (**B**). KRT80 expression was assessed by normalizing the data based on the housekeeping gene Gapdh values. Graphs are based on the mean ± SEM; * *p* < 0.01.

The *Krt80* gene expression levels in the medial and distal colon portions were found to be significantly lowered in the miR-195-5p-treated mice versus the vehicle control group (*p* < 0.01; Figure 5).

**Figure 6:**
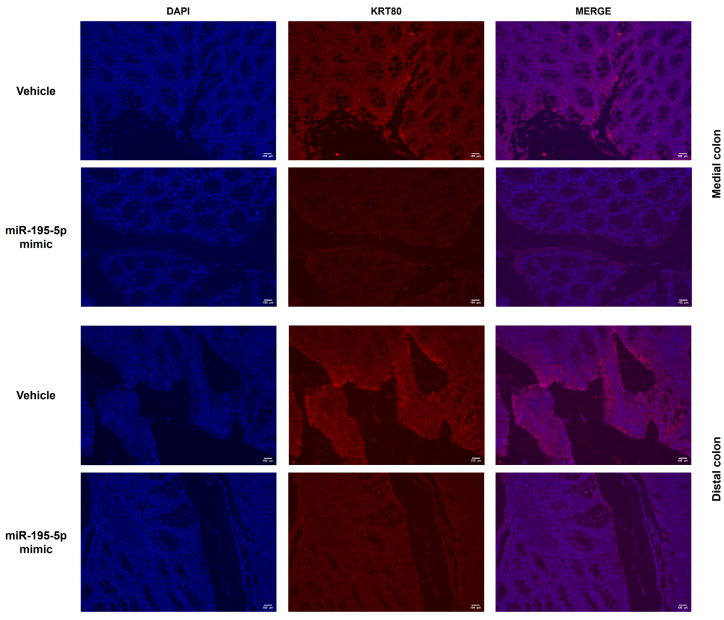
In vivo miR-195-5p impact on KRT80 protein expression. KRT80 protein expression was investigated in the medial and distal colon of AOM/DSS mice (*n* = 14 mice/group). The administration of miR-195-5p decreased the KRT80 protein levels, which corresponds to the gene expression data. Representative images of DAPI and KRT80 signals were acquired and merged. Original magnification: 20×.

In addition, using the same mice colon samples, we assessed the KRT80 protein expression. Our results showed a greatly decreased KRT80 level in both colon segments of the treated mice (Figure 6), confirming the potential impact of miR-195-5p and KRT80 on tumorigenesis.

### 3.5. KRT80 Knockdown by Small Interfering RNAs (siRNAs) in CRC Cell Lines

**Figure 7:**
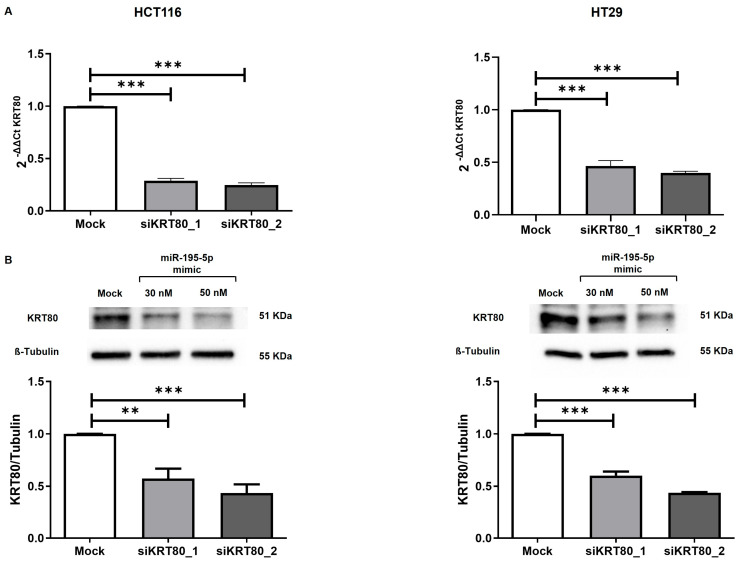
KRT80 knockdown in CRC cells by siRNAs. (**A**) RT-PCR analyses demonstrated the effect of two different siRNA transfections on KRT80 mRNA expression in the HT29 and HCT116 cell lines. Relative KRT80 expression was normalized based on the GAPDH control (*n* = 4, mean ± S.D); *** *p* < 0.0001. (**B**) WB assay showed a significant suppression of KRT80 expression in both transfected conditions. Detection of ß-tubulin expression was performed to monitor protein loading and to normalize the KRT80 values (n = 4, mean ± S.D); ** *p* < 0.001; *** *p* < 0.0001.

To further evaluate the functional significance of KRT80 in CRC, we carried out knockdown studies using specific siRNAs for targeting the KRT80 mRNA. The inhibitory properties of two independent siRNAs (siKRT80_1 and siKRT80_2) specific to KRT80 were investigated in the HCT116 and HT29 cells lines. The transient transfection of each siRNA provided effective suppression of the KRT80 mRNA in all the cell lines (*p* < 0.0001; Figure 7A).

In regard to the transfected conditions of each siRNA, we also assessed the KRT80 protein expression, finding a remarkable downregulation compared to the mock control in both the HCT116 and HT29 cell lines (*p* < 0.001; *p* < 0.0001; Figure 7B).

### 3.6. Effect of KRT80 Downregulation by miR-195-5p or siRNA on CRC

The KRT80 functional interaction network was investigated to study its oncogenic potential through the use of the GeneMANIA algorithm, which revealed a strong physical interaction between KRT80 and CCND1 (cyclin D1) (Figure S1).

**Figure 8:**
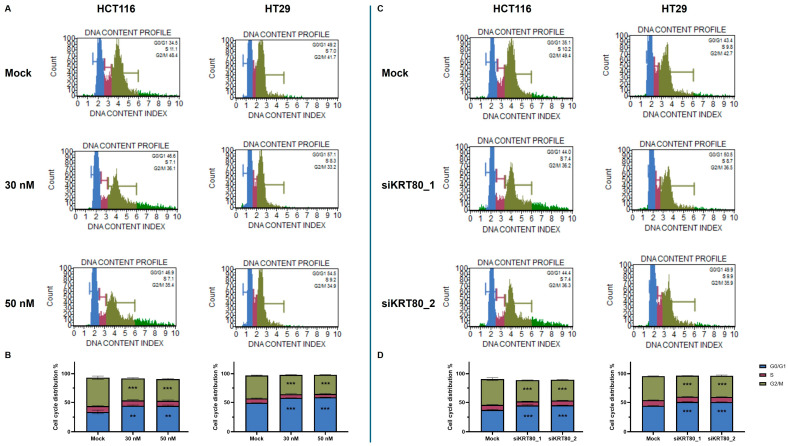
Effect of KRT80 regulation on cell cycle in CRC. (**A**,**B**) Cell cycle progression in transfected cells was influenced by miR-195-5p compared to the mock control in both cell lines, highlighting G0/G1 cell cycle arrest. (**C**,**D**) Knockdown of KRT80 by two siRNAs suppressed the transition of HCT116 and HT29 cells from the G1 to S phase, reducing the G2/M cell population. Cell cycle distribution values are reported in Table S2. Data derived from at least 3 independent experiments and expressed as mean ± SEM; ** *p* < 0.001; *** *p* < 0.0001.

In addition, a recent study reported that KRT80 knockdown affected the cell cycle activity via CCND1 expression in lung adenocarcinoma cells [10]. Based on this evidence, we performed transfections with miR-195-5p mimic or siRNA molecules to evaluate whether this could change the downstream biological effects on the cell cycle and their effect on CRC growth. We assessed the cell cycle profiles of synchronized and transfected cells using flow cytometry and found that miR-195-5p significantly increased the proportion of cells arrested in G_1_, concomitantly with reductions in G_2_/M populations (*p* < 0.001; *p* < 0.0001; Figure 8A and Table S2). Likewise, siRNA-mediated knockdown of KRT80 revealed a significant change in the cell cycle distribution, with a pronounced G_1_-phase block and a decrease in the number of cells in the G2 phase (*p* < 0.0001; Figure 8B and Table S2).

## 4. Discussion

Cell–cell communications are essential for maintaining homeostasis, facilitating growth, immune responses, tissue repair, and coordinating complex biological processes [30]. The role of keratins is critical for ensuring intercellular adhesion and for triggering various intracellular signal transduction cascades [1]. KRT80 is a keratin intermediate filament that provides cytoskeleton integrity, mechanical support, and protects cells from stress. Many studies have investigated the role of KRT80 in tumors, focusing on its regulatory functions in several networks that have an impact on cancer cell mechanisms and on patient outcomes. Recent evidence shows that KRT80 is aberrantly expressed in a variety of malignancies, including colorectal, gastric, lung, ovarian, and hepatocellular carcinomas [31]. The overexpression of KRT80 in these cancers has been correlated with more aggressive tumor phenotypes, higher rates of proliferation, enhanced migratory and invasive capabilities, and poorer clinical outcomes [31]. For instance, in ovarian cancer, KRT80 overexpression promotes cell proliferation, invasion, and migration, and is associated with unfavorable patient prognosis [7]. In hepatocellular carcinoma, KRT80 functions as an oncogene, influencing the epithelial–mesenchymal transition and modulating the PI3K/AKT signaling pathway, thereby contributing to tumor progression [9]. A recent study [10] showed that KRT80 is markedly overexpressed in lung adenocarcinoma, correlated with poorer patient outcomes and enhanced G_1_-to-S progression in lung cancer cell lines. These observations have raised important questions regarding the functional role of KRT80 in tumor biology and its potential utility as a prognostic biomarker and/or therapeutic target. Although KRT80 may act as a mediator of oncogenic signaling with a potential impact on tumor behavior, the precise molecular mechanisms underlying its contribution to CRC development require further investigation.

Over the last few years, the regulatory role of microRNAs (miRNAs) in gene expression at the post-transcriptional level, along with their involvement in the development of various diseases [32,33,34,35,36], including colorectal cancer (CRC) [37,38,39], has raised significant research interest, particularly owing to their potential therapeutic applications. Some miRNAs have been identified as key regulators of keratin expression, playing a critical role in cancer pathogenesis. For instance, miR-485-5p has been shown to downregulate KRT17, a keratin associated with aggressive tumor behavior, thereby suppressing tumor growth in gastric cancer, both in vitro and in vivo [20]. Similarly, KRT80 regulated by the miR-206/ETS1 axis plays a pivotal role in promoting tumor progression in ovarian cancer through the activation of the MEK/ERK signaling pathway [7]. Moreover, other studies have reported that circular RNAs or lncRNAs modulate KRT80 expression via sponging miRNAs in gastric and colon cancers, reinforcing the broader relevance of the post-transcriptional regulation of KRT80 in epithelial tumors. Recent evidence in regard to gastric cancer shows that circPIP5K1A functions as a competing endogenous RNA by sponging miR-671-5p, thereby relieving the repression of KRT80 and activating PI3K/AKT signaling to promote tumor progression [40]. In CRC, LINC01485 is upregulated and promotes tumor cell proliferation, migration, invasion, and in vivo growth, by acting as a ceRNA for miR-383-5p. By sponging miR-383-5p, LINC01485 relieves the repression of KRT80, leading to elevated KRT80 levels; the knockdown of LINC01485 reduces malignancy phenotypes, which are rescued by miR-383-5p inhibition or KRT80 overexpression [41].

In our previous works [16,29], we have characterized the effect of miR-195-5p on the regulation of γ-catenin, a desmosome component that is significantly involved in CRC cell proliferation, viability, migration, and invasion, via Wnt signaling. Additionally, the in vivo administration of miR-195-5p significantly decreased the number of malignant lesions in the colon in a CRC mice model and effectively inhibited tumor growth [29]. We have also highlighted the impact of miR-195-5p on the desmosomal complex, identifying a novel interaction between miR-195-5p and PNN, a desmosome-associated protein that plays a critical role in CRC development, which was able to enhance the strength of cellular adhesive junctions through the upregulation of KRT8 and KRT19 [21].

Furthermore, we have recently demonstrated the effect of miR-195-5p on CRC progression in controlling the attach/detach potential, the migration/invasion abilities, the relative proliferation rate, and the apoptotic process by regulating KRT23 expression [22]. We have also observed that the in vivo delivery of the miR-195-5p mimic reduced the KRT23 levels in AOM/DSS mice [22].

In the current study, with the aim of further investigating the miR-195-5p-guided mechanisms in the modulation of keratin filaments, we performed a bioinformatic analysis, which revealed two potential binding sites in the 3′UTR of the KRT80 transcript for miR-195-5p. Subsequently, we analyzed the KRT80 expression in human normal and cancerous tissue, exploiting public data repositories. To compare and validate these data, we assessed KRT80 mRNA and protein expression in paired CRC and non-tumor tissues from CRC patients collected at our hospital, highlighting a critical overexpression in the tumor portions. We have also explored the biological impact of miR-195-5p on KRT80 expression, demonstrating that the in vitro overexpression of miR-195-5p resulted in a substantial reduction in KRT80 mRNA and protein levels. In addition, to define the effectiveness of the gain in miR-195-5p on KRT80 levels we evaluated its expression in the colon portions of miR-195-5p-treated mice, showing markedly decreased expression compared to the AOM/DSS control group.

The subsequent knockdown of KRT80 in CRC cell lines was assessed using siRNAs at the mRNA and protein levels to validate its oncogenic potential through its interaction with CCND1. This demonstrated that the loss of function of KRT80 affected the cell cycle activity, inhibiting the transition of CRC cells from the G1 phase to the S phase and, hence, tumor growth.

Collectively, our findings further emphasized the potential abilities of miR-195-5p, revealing a novel regulatory axis with keratin intermediate filaments in CRC. Nevertheless, additional research is required to elucidate in greater depth the molecular mechanisms associated with KRT80 in CRC progression. Moreover, miR-195-5p-based strategies in colorectal cancer hold significant therapeutic promise. However, challenges remain, including in regard to efficient miRNA delivery, its integration with conventional drugs or biological agents, and managing potential cytotoxicity [42,43]. The potential cytotoxicity and immune responses triggered by both the miRNA cargo and its delivery vehicle require rigorous preclinical dose escalation and repeated administration studies involving appropriate experimental models [44]. Transcription factor decoy oligodeoxynucleotides may enhance the control over gene expression by sequestering key regulators upstream of transcription [45,46]. Future research must focus on optimizing delivery vehicles and unraveling complex biological interactions to achieve safe and effective combination therapies.

Furthermore, considering the widespread dysregulation of KRT80 and the emerging evidence supporting the tumor-suppressive role of miR-195-5p in multiple cancer types [31], this therapeutic strategy holds strong potential for broader oncological applications, extending beyond colorectal cancer to other malignancies characterized by similar molecular alterations.

## 5. Conclusions

In summary, this study provides evidence that KRT80, a crucial element of keratin intermediate filaments, is significantly overexpressed in CRC patients. Interestingly, while cytokeratins are traditionally associated with epithelial differentiation and structural stability, recent evidence suggests that certain keratins, such as KRT80, may acquire pro-tumorigenic roles in specific contexts. Moreover, miR-195-5p effectively restores normal KRT80 expression in CRC cell lines and AOM/DSS-treated mice, highlighting its regulatory role. Additionally, the critical oncogenic properties of KRT80 were demonstrated, both siRNA- and miR-195-5p mediated KRT80 repression, impairing tumor growth by inhibiting cell cycle progression. These findings further reinforce the clinical potential of miR-195-5p in affecting keratin expression and influencing CRC progression.

## 6. Patents

An Italian patent entitled “MiRNA or combination of miRNAs for therapy” was issued on 4 June 2024 to the Ente Ospedaliero Specializzato in Gastroenterologia “Saverio de Bellis”.

## Data Availability

The data are contained within the article and Supplementary Materials. The raw data from the Western blot experiments presented in the study, are openly available in FigShare at doi 10.6084/m9.figshare.29126777.

## Supplementary Materials

The following supporting information can be downloaded at: https://www.mdpi.com/article/10.3390/cancers17132183/s1, Figure S1. KRT80 interaction through Genemania tool. Table S1. List of miR-195-5p gene target predictions. Table S2. Percentage of cell cycle distribution in all conditions with and without miR-195-5p or KRT80.
