# Supplementary material for: miR-195-5p Suppresses KRT80 Expression Inducing Cell Cycle Arrest in Colon Cancer
Source: Cancers (Basel). 2025 Jun 28;17(13):2183. doi: 10.3390/cancers17132183 (PMC12248558; doi:10.3390/cancers17132183)
Supplement: Supplementary file 1 [file cancers-17-02183-s001.zip › Table S2.pdf]

	HCT116			HT29		
**Cell cycle phase**	**G0/G1**	**S**	**G2/M**	**G0/G1**	**S**	**G2/M**
**Mock (% of cells)**	33.0	9.0	50.0	49.0	7.0	41.0
**30 nM (% of cells)**	45.0	8.0	37.0	58.0	7.3	33.0
**50 nM (% of cells)**	45.0	7.6	36.0	59.0	7.0	33.0

	HCT116			HT29		
**Cell cycle phase**	**G0/G1**	**S**	**G2/M**	**G0/G1**	**S**	**G2/M**
**Mock (% of cells)**	36.0	9.5	48.0	44.0	9.5	43.0
**siKRT80_1 (% of cells)**	45.0	7.0	36.0	51.0	9.0	36.0
**siKRT80_2 (% of cells)**	46.0	7.0	36.0	51.0	9.0	35.0
