# Supplementary figures and images for: miR-195-5p Suppresses KRT80 Expression Inducing Cell Cycle Arrest in Colon Cancer
Source: Cancers (Basel). 2025 Jun 28;17(13):2183. doi: 10.3390/cancers17132183 (PMC12248558; doi:10.3390/cancers17132183)

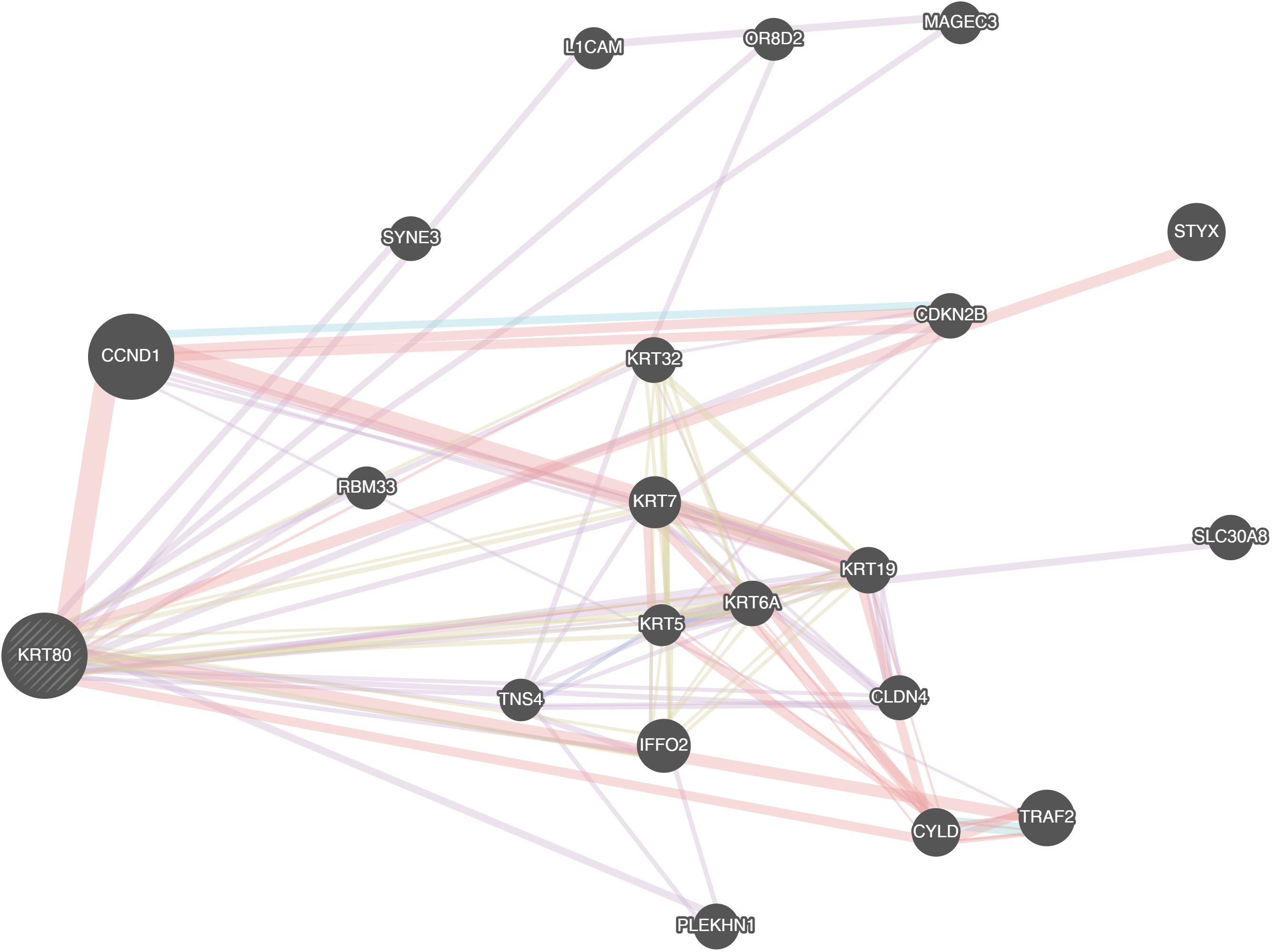

Supplement: Supplementary file 1 [file cancers-17-02183-s001.zip › Figure S1.jpg]
